# “I was forced into it”: The continued violation of widows from the Luo community of Kenya through sexual cleansing rituals

**DOI:** 10.3389/fgwh.2022.942635

**Published:** 2022-08-16

**Authors:** Leso Munala, Esther Mwangi, Margaret Harris, Nene Okunna, Bethlehem Yewhalawork, Maureen Ong'ombe

**Affiliations:** ^1^Department of Public Health, St. Catherine University, St. Paul, MN, United States; ^2^Department of Health Studies, Saint Joseph's University, Philadelphia, PA, United States; ^3^Independent Researcher, Nairobi, Kenya

**Keywords:** sexual cleansing, cultural practices, widow inheritance, social construction, Kenya

## Abstract

Sexual cleansing is part of the social transition process for widows to become eligible to remarry after the death of her husband. This ritual is conducted to cleanse the widow of evil spirits resulting from the death of her husband. This qualitative study explored the sexual cleansing ritual in the Luo community in southwest Kenya. This paper aims to examine the widows' perceptions of the social constructs surrounding the practice of the sexual cleansing ritual that maintains its continued existence in this community. Twenty-seven face-to-face in-depth interviews were conducted with widows who had undergone sexually cleansing. Data was analyzed using conventional content analysis. Three main themes emerged in the exploration of the social construction of the sexual cleansing ritual of widows. The findings therein highlight the precarious situation of widows and the need for social support services for women who have been sexually cleansed.

## Introduction

Widowhood not only symbolizes the loss of a partner through death but also the departure of a breadwinner and a shift in the woman's lifestyle and social status in the community (1). In many societies in Africa and Asia, widowhood is considered a bad omen, leading to the social alienation and stigmatization of widows and can be remedied through various rituals (2, 3). The widow, and rarely the widower, bears the responsibility to carry out the purification rituals upon the death of the spouse (2, 4). Widows are required to undergo widowhood rituals to eliminate the impurity of their status and sexual cleansing is one of such cultural rituals (5). Available literature provides documentation of the negative emotional and psychological consequences associated with widowhood due to the ill-treatment they receive from their communities (6, 7).

According to Ayikukwei et al. (8), sexual cleansing is a cultural practice of social transition and is characterized by three stages identified as separation, transition, and incorporation. The purpose of sexual cleansing is to reintegrate the widow back into the community, and to mark the transition from being removed from social life to the acceptance into communal life (8, 9). The practice of sexual cleansing indicates that the widow is eligible for remarriage through wife inheritance (1, 8, 9). This cultural practice is considered a rite of passage to wife inheritance which allows for the continuation of the family as these new unions can produce children to carry the family name from observing sexual cleansing (8). The widow is then “owned” by a male relative of the deceased who provides her companionship and assistance after the death of her husband (10–12). Variations in the sexual cleansing ritual are observed in several communities in Eastern and Southern African countries such as the Luo in Kenya (13), Nkore ethnic group in Uganda (14), Aushi in Zambia (15), Zimbabwe, eastern and southern regions of Malawi (16), and central and southern parts of Mozambique (4). In Kenya, sexual cleansing, known as tero chi liel in Dholuo, is practiced within the Luo ethnic community (10–13). The practice involves unprotected sexual activity between the widow and a male relative of the deceased or a hired cleanser (4, 9, 11, 12). The effectiveness of the cleansing is determined by the strict compliance to the rituals such as the number of days, time of day, and location of the sexual intercourse to change the widow's social status (4).

These rituals often stem from how various societies understand and make meaning of death (1, 17). In some societies, the meaning and traditions that encompass death are opportunities to express grief and show respect to the deceased (1, 17, 18). Among the Luo, one of the myths is the existence of supernatural spirits of the deceased i.e., that the spirit remains among the living and plays an active role among the living day-to-day lives (8). It is believed that the death of the husband is evil and thus makes the widow impure. Her state of impurity is considered a source of misfortune for her and her children as well as other members of the community (4, 8, 10, 11, 13). To be liberated from this evil, widows are subjected to sexual cleansing, a purification ritual that requires unprotected intercourse as semen is perceived to be the cleansing agent (8). Additionally, undergoing this ritual protects members of the homestead from further misfortune such as illness, locally known as chira in Dholuo (19).

Sexual cleansing is often carried out by a male in-law relative, usually the brother-in-law. Hired cleansers and non-relatives are at times sought for this purpose because of the unwillingness of the in-laws to economically support the widow (8–12). A hired cleanser is a non-relative who is paid for the act of sexually cleansing the widow (9). In cases where a hired cleanser is used, it is for the purposes of cleansing from evil spirits and is not connected to financial or parenting obligations. The ritual has been commercialized to give monetary incentives due to the HIV epidemic in Western Kenya, and this has created a demand for hired cleansers (9). Available research shows that the disproportionate burden of HIV among women within the Luo community is strongly associated to sexual cleansing (4, 9, 11, 12, 20). An HIV-positive hired cleanser engages in concurrent sexual partnerships, transmitting HIV to other widows in the community (11, 12). Further, according Kenya HIV estimates report 2018, Siaya County (study area) had an HIV prevalence of 21%, approximately four times the national prevalence of 5.9% (National AIDS Control Council, 2018).

### The social construction and perpetuation of sexual cleansing

Sexual cleansing is a social norm of widowhood in some cultures. Social norms are created, understood, and exchanged through social interactions among members of a family, community, and society. Social norms govern the behavior of people of the same reference group through social interactions (21). Through social interactions, social knowledge is understood and exchanged to create a sense of reality that becomes embedded into the institutions of society, and it is enacted based on the reciprocal interactions of people's roles in relation to each other. The social realities are created to lead to the understanding of the intersubjective set of beliefs and behaviors that are considered true and appropriate to define the individual's concept of reality (22).

Cultural practices such as child marriages, Female Genital Cutting/Mutilation (FGC/M), breast ironing, foot binding, and female infanticide and/or son preference are among several cultural practices that perpetuate violence against women and girls. These cultural practices persist even though most practicing communities are aware of the psychological and health consequences. For instance, FGC/M is internationally recognized as a violation of human rights (United Nations, 2013), but often justified as a protective factor to uphold and maintain family and community values of honor, marital fidelity, spiritual cleanliness, and premarital virginity (23, 24). The associated meanings and values are how cultural practices are socially constructed and reinforced through social pressure and social obligation within the community. Sexual cleansing is a social construct because its meaning is created through social interactions guiding people's behavior and actions in accordance with widely agreed-upon norms. Widows are compelled to engage in sexual cleansing to conform to societal norms of behavior. Research shows that informal social sanctions are usually placed on the widow by family and community members if she refuses to undergo the cleansing ritual because it is the accepted pre-requisite to reintegrate her back into the community (4, 8, 11, 12). Also, those who refuse to undergo sexual cleansing are ostracized, taunted, and humiliated (8). These social sanctions are in place because of the traditional perceptions in the Luo community that a widow possesses evil spirits as a result of the death of her husband (4, 8, 9). To say that sexual cleansing is socially constructed is to emphasize that the meaning, idea, and understanding of the cultural practice are shaped by social forces of human interactions for the practice to exist and evolve over time and be maintained in the community. The meanings and interpretations of this ritual remain a powerful driving force for the widows as well as the members of the communities they are a part of.

This paper aims to examine the widows' perceptions of the social constructs surrounding the practice of sexual cleansing ritual that maintains its continued existence in the Luo community.

## Methods

### Study location

The study was carried out in Siaya County, one of the 47 Counties in Kenya. It is a mostly rural County with a land surface area of ~2,530 km^2^, bordering Lake Victoria in Western Kenya. It comprises of six sub- counties namely: Alego Usonga, Bondo, Ugenya, Ugunja, Gem, and Rarieda (25). The participants for this study hailed from Ugenya Sub County. The main economic activities in Siaya County are fishing, small scale trading and subsistence farming.

### Study participants

Participants were selected through criterion sampling in which the researchers purposefully identified and recruited the widows. To be eligible to participate in the study, participants had to be a widow, 18 years or older, reside in Siaya County, be from the Luo ethnic group, and had undergone sexual cleansing. In total, 27 face-to-face in-depth interviews were completed in Dholuo and Swahili with widows aged 29–90 years old, who had undergone sexual cleansing. All participants resided in Ugenya sub- county in Siaya County, the predominant location of the cultural practice of sexual cleansing.

### Data collection

This study used a qualitative research study design. The protocol utilized a semi-structured interview format with 10 main questions with probes and demographic information that was collected at the conclusion of the interview. Questions for the interview protocol were generated based on a review of the literature on sexual cleansing and wife inheritance practices. The questions focused on the experience of widowhood, challenges faced, community treatment, reasons for sexual cleansing, and perceptions of sexual cleansing. The interviews were conducted by three members of the research team, two of whom were from the Luo ethnic community and fluent in both Dholuo and Swahili. Ethical approval to conduct this study was obtained from University institutional review board (IRB) and Amref Health Africa Ethics and Scientific Review Committee (ESRC). Participants signed an informed consent that explained the purpose of the research, procedures, and confidentiality of the information shared prior to starting the audio recording. The interviewer verbally shared the purpose of the research, procedures, and confidentiality of the information that will be shared prior to the start of the interview. Participants were also given the opportunity to ask any questions before starting the interview. All interviews were digitally recorded with the permission of the study participants and transcribed verbatim by two Dholuo members of the research team.

### Data analysis

The conventional content analysis technique was applied for data analysis. Conventional content analysis is considered ideal for the analysis of data to gain a richer understanding of a phenomenon being studied and is considered appropriate for this study because current literature on sexual cleansing is limited (26). Additionally, this qualitative approach to analysis was most appropriate because the interview protocol questionnaire and probes were open-ended to allow researchers to gain direct information without imposing preconceived categories (26). As most of the interviews occurred in Dholuo and not all investigators were native speakers, the co-investigator conducted quality checks in transcripts where further clarification was needed to confirm that the perspectives of the participants were reflected in the data. The initial analysis included three of the five authors reading three to five transcripts at a time to note patterns, themes, outliers, and interesting aspects of the interviews. This process allowed for codes to emerge from the data through an iterative process of reading and re-reading the transcripts. The coding scheme was developed by the aforementioned research team who met weekly for 3 months to share impressions and brainstorm themes and patterns to develop a coding scheme. The coding scheme included codes organized into categories based on similarities and differences of each parent and child code. The team also defined each code which was then transferred to Dedoose software, to run coding reports for each code. The reports were analyzed for trends, patterns, occurrences of codes, and emerging themes. The creation of memos for each report to further explore and summarize the findings for each code across the transcripts was completed. The memos were then utilized to further review and analyze the themes, how themes fit together and then relate to the larger sociocultural context.

## Results

A total of 27 widows participated in this study. The three main themes connected to the social construction of sexual cleansing were: (a) Reasons for the practice: to remove impurity, to protect the children, to protect the homestead, and to uphold tradition; (b) Widows attitudes of the practice: community treatment, to be eligible to remarry and; (c) enforcement of the practice: role of the widow's family, the role of in-laws, and role of the children. The demographic characteristics of the participants are displayed in Table 1.

**Table 1:** Demographic description of the study participants.

**Participant no**.	**Age**	**Education level**	**No. of children**	**Sex of children**	**Occupation**
1	58	Standard 4	7	3 Male, 4 Female	Unemployed
2	53	Standard 7	7	5 Male, 2 Female	Unemployed
3	62	Standard 6	1	1 Male	Farming
4	48	Completed primary school	5	3 Male, 2 Female	Unemployed
5	66	Some primary school	6	1 Male, 2 Female	Unemployed
6	71	No formal education	4	1 Male, 3 Female	Unemployed
7	29	Some secondary school	2	1 Male, 1 Female	Domestic maid
8	46	No formal education	6	4 Male, 2 Female	Unemployed
9	69	No formal education	9	3 Male, 6 Female	Unemployed
10	40	Some primary school	4	1 Male, 3 Female	Unemployed
11	48	Standard 7	7	2 Male, 5 Female	Unemployed
12	62	No formal education	7	4 Male, 3 Female	Unemployed
13	48	Some primary school	5	2 Male, 3 Female	Unemployed
14	46	Standard 8	6	4 Male, 2 Female	Farming
15	60	No formal education	4	1 Male, 3 Female	Unemployed
16	66	Standard 6	8	2 Male, 6 Female	Unemployed
17	39	Standard 8	4	1 Male, 3 Female	Unemployed
18	73	No formal education	9	2 Male, 7 Female	Unemployed
19	65	Standard 4	6	2 Male, 4 Female	Charcoal trader
20	70	No formal education	5	2 Male, 3 Female	Unemployed
21	38	Some primary school	3	3 Male	Unemployed
22	35	Standard 6	3	3 Female	Street hawker
23	37	Some secondary school	3	1 Male, 2 Female	Teacher
24	90	No formal education	2	2 Female	Unemployed
25	32	Standard 7	5	2 Male, 3 Female	Street hawker
26	37	Some primary school	6	6 Female	Unemployed
27	32	Some primary school	2	1 Male, 1 Female	Unemployed

### Reasons for the practice

All of the participants described sexual cleansing as a widely known practice within the community. The reasons for the practice was defined as participants explaining the ritual and the purpose of sexual cleansing. A majority of the participants mentioned sexual cleansing removes impurity ascribed to her, protects the children, and homestead, and upholds tradition.

#### To remove impurity

Sexually cleansed widows reported their knowledge of the practice when asked “what are some of the reasons why a widow is sexually cleansed in this community?” A majority of the participants mentioned sexual cleansing removes impurity or evil to protect the children, extended family, community, and homestead. Every widow mentioned that she was perceived in the community as having a bad omen because of the death of her husband. A widow must be sexually cleansed to remove bad omen, and sex must be part of sexual cleansing. Only one participant mentioned that cleansing should be done publicly so that community members know that the widow has been rid of a bad omen. If the widow is not cleansed, she cannot visit other people's homes, mingle freely with others, collect water from the river, and eat with her children as illustrated in the quote below:

“*It would be said a widow has bad omen - you cannot visit other homes, collect water from the river, go to someone's farm because the crops will wither. So, they need to be cleansed.” (Participant 3)*

“*In those days I could not even be hired to work on somebody's farm. If I went, I would be asked why I had gone to someone's farm before I was cleansed from a bad omen.” (Participant 20)*

#### To protect the children

All widows mentioned that they were sexually cleansed to protect their children. Widows were sexually cleansed to protect the children from death and illness. Most of the widows believed that the cause of diseases like Malaria and other misfortune that result in death is caused by a mother who is not sexually cleansed. Sexual cleansing protected the children from illness and death as illustrated below:

“*Among the Luo's, a widow is cleansed to remove bad omen[impurity] in the home. Also, to prevent death in the home. I was cleansed and fulfilled all the requirements and yet my daughter still died. They told me to be cleansed to protect my daughter and yet she died in a road accident.” (Participant 7)*

“*But if she refuses and she is left and her child suffers from illness like Malaria, it is said it is because she was not cleansed.” (Participant 27)*

Participants described protecting the children, especially sons because they are valued more than daughters, and it was an obligation that must be fulfilled. Before the widows were sexually cleansed, they were told by community members including extended family members that protecting the children meant not cooking, eating, feeding, living in the same house, touching or caring for them. If the widow is not cleansed, she cannot enter other people's homes, mingle freely with others and eat with her children.

“*It would be said a widow has bad omen [impure] - you cannot visit other homes, collect water from the river, go to someone's farm because the crops will wither. So, they need to be cleansed.” (Participant 1)*

All of the participants explained that sexual cleansing must occur so that the widow can be with her children.

“*That it [sexual cleansing] would help the children. If you are not cleansed and you eat with the children after your husband dies, they will be affected negatively.” (Participant 14)*

#### To protect the homestead

One of the key findings was that sexual cleansing protects the homestead, and more than half of widows were cleansed because they needed a new house structure built. The homestead included children, in-laws, and other extended family members. Sexual cleansing protects the homestead and is performed in a grass-thatched house. A grass-thatched house was described as a temporary structure where the widow lived for a short time and then transitioned to a permanent structure. The temporary structure was built to abandon the main house to rebuild again, this was referred to as *changing the house*. According to traditions, a widow was prohibited by in-laws to move from a grass-thatched house to a permanent structure unless she was sexually cleansed. The transition to a grass-thatched house was initiated when the widow was denied access to the main house, where she lived with her husband. To build a permanent structure, the widow needed the help of a brother-in-law or paid a mason, and those who paid reported financial challenges. The man cleansing the widow must build her a new house.

“*You are told that if your house is not in a good condition, you cannot build another house on your own. You can only build a new one with a brother in law.” (Participant 23)*

“*So, the problem of not having a house made me look for a brother-in-law because my husband's relatives were not willing to help me build a house because I was a widow. So, you have to look for a brother in-law to cleanse you to build you a house.” (Participant 6)*

“*The change that has been there – after my husband died I had a small grass thatched house like that one. Then the man who cleansed me built me a better house – a grass thatched one.” (Participant 5)*

An uncleansed widow cannot stay in the same home with her children because she is blocked from accessing her house. A few widows described living in poor housing conditions where the house was too small and/or the roof had holes and would get rained on with her children. The grass-thatched house cannot be repaired and the widow cannot move out if she is not sexually *cleansed*.

#### To uphold tradition

Eighteen of the widows mentioned that they were sexually cleansed to fulfill Luo traditional requirements. The demands prescribed to the widow to uphold tradition is one of the main reasons why sexual cleansing occurs.

“*The main reason why a widow is cleansed is because of Luo traditions. Sometimes when a daughter wants to get married, there should be a man living in the widow's house. If a son wants to marry or build a house, his widowed mother should be living with a man in her house. A widow also needs a man to set up a home. I think those are some of the reasons why widows get cleansed. If it was not for these demands, there would be no need for cleansing to be done” (Participant 23)*

Some participants explained that following traditions was dependent on if the widow was married into a family that practiced sexual cleansing where other women from the husband's lineage were also cleansed. If the family believes in sexual cleansing, the widow must be cleansed. Four participants noted that they were sexually cleansed because other women in the family were sexually cleansed.

The tradition is considered complete when the cleanser has sexual intercourse with the widow for 4 days in a grass-thatched house. If the cleansing is not considered complete and the cleanser has spent the night in the grass thatched house, a new grass-thatched house must be built for another cleanser to complete the ritual. Sexual intercourse is a required component to fulfill this tradition, the cleanser cannot just sleep next to the widow. The following excerpt describes the required sexual component of the practice:

“*There is no other way. For the Luo's sex is attached to so many traditions. If there is no sex, the activity is considered null and void. For example, if a man is helping you set up a home and there is no sex, the home has not been set up in the required way. Sex is an integral part of Luo traditions.” (Participant 19)*

The sexual activity must occur at night, not during the day and the cleanser must ejaculate for the tradition to be complete. If the deceased had multiple wives, the cleanser alternates between the different houses to cleanse all the wives on the same night. To some, the hair of the widow, either the pubic area or head must be shaved by an elderly woman or sister-in-law to show that she has followed tradition. The shaving of the hair presumably takes the ghost/spirit of the deceased away.

“*You have sexual intercourse with the man cleansing you. The following morning you take your clothes to throw them away near the river. An old lady who cooked for you when your husband died comes to shave your hair. After she has shaved your hair, you go with her to your parents' home to take the ‘shadow.' A goat is slaughtered for you and you carry the meat back to your husband's home*.” *(Participant 15)*

Similarly, another widow narrated that after she has been cleansed, she had to go to her parent's home and return with a hen to prepare and serve for the cleanser to eat with her children.

“*The family had a meeting to discuss my cleansing. After I cleansed according to Luo traditions I was told to go back to my parents' home, spend a night and come back with a hen which is given to the man who cleanses you. I did that and came back with a hen. That is how I was cleansed.” (Participant 17)*

A few women noted the financial challenges to pay a cleanser to uphold the tradition because cleansers are expected to be paid for their service. Men who demanded and received monetary value were not related to the widow or the deceased family, but rather community members whose profession was solely to cleanse widows in the community. Below are excerpts to illustrate the financial burden placed on the widow to fulfill the traditional requirements:

“*I paid him, so that he could help me meet the traditional requirements to enable me go back to my house. Before that I had not found the money and my mother also died. When I went to our home where I was born I was not allowed to enter the home because I had a bad omen.” (Participant 11)*

“*The practice of cleansing is difficult. It is not a good thing. It is a bad thing. Although we were forced because it is a Luo custom that requires it to be done. It is not an easy thing. It is costly because if you do not have money you cannot do it. The men who cleanse want to be paid. It is not an easy thing. It is tough. It brings a lot of problems in the home.”(Participant 18)*

### Widow's attitudes of the practice

The widow's own attitudes about sexual cleansing was influenced by community members. Even though all the widows perceived sexual cleansing as outdated, they participated in the practice because of community treatment and the eligibility to remarry.

#### Community treatment

All participants described being treated differently because their husband had died. Multiple participants mentioned that community members regard the woman as a useless person once widowed. The term ‘useless person' was used to refer to being perceived as a ‘nobody' by community members after the death of her husband. She is only respected if she is somebody's wife.

“*When my husband was there, they respected me. People could not talk to me carelessly. After my husband died, people talk to me carelessly. They despise me because I do not have someone to support me. If you have a problem, you cannot share. You can cry because you have been told something that hurts you. So that caused me a lot of pain after my husband died.” (Participant 13)*

Participants reported that other forms of treatment include being physically beaten by in-laws and other community members, and her children can also receive the beating. Some explained that widows are abused, despised, and disrespected because she is a widow. The treatment also applies to her children as quoted below:

“*No one can come and abuse a married woman, no one can come and tell her “I want to take your tree” no one can come and cane her child and tell her that “I found your child had teased my child” A widow's child can be beaten because their father died. A married woman is not disrespected. Because it is said, will you harass her and her husband is there? Will her husband let you harass her? But a widow is like trash. Anyone can say whatever they want to say about her.” (Participant 4)*

“*The community shun her. You are not wanted. You are considered stubborn, strong headed. Someone who does not respect traditions.” (Participant 6)*

The main difference between the treatment of a married woman compared to a widow is that people had no respect for widows, they insult her, make false accusations, as well as take her household belongings, and destroy her cultivated land. One participant gave an example that community members will intentionally destroy her crops by letting cows loose on her farm.

“*Challenges are many – you are despised because you are a widow. People provoke you. Someone will let cows loose into your farm to destroy your crops and you cannot protest. They will not even apologize. Even if you protest, there is nothing you can do to the person.” (Participant 6)*

Three participants described that her in-laws persistently avoided her in person and/or over the phone because they think that she is asking for money as they know that she is alone and had nobody for support.

“*Yes, there is a difference. There is a difference because when your husband dies, the family members shun you. They say that when you go to see them or your brother in law or son who comes to visit that you are going to beg them for money especially in these times when people are very selfish. So, people avoid you.” (Participant 18)*

Only one participant mentioned that because of her status as a widow, she has the freedom to do things that she has planned because she doesn't have to explain her intentions to a husband like a married woman. The same participant did not think there was a difference in treatment and status of a widow compared to a married woman because regardless, they have the same problems. The majority of the participants mentioned that a married woman receives support and has somebody to share and resolve problems, unlike a woman whose status is a widow. The difference in the treatment between how a married woman and a widow is evident by how they are treated by men and other women in the community, specifically married women, sisters-in-law, and mothers-in-law.

Some participants explained that sources of conflict and negative treatment from other women in the community is because men who cleanse have wives, some of whom do not agree with the practice. Two participants stated that married women consider widows a threat to their marriage because they believe that the widow will snatch their husbands away from them. If seen talking to men, the widow is falsely accused of having an affair and is a source of tension between community members.

One participant also stressed the fact that men in the community do not want their wives interacting with the widow because she is viewed as having ‘loose morals.' All 27 participants described their experiences about how they are perceived by community members as having a restricted life in their interacting with other people.

Types of restrictions placed on an uncleansed widow include not fetching water from the same stream, eating with others, sitting on designated chairs, and not entering other people's homes thus limiting her interactions with other community members. Several participants mentioned that a widow cannot enter other people's homes unless she is cleansed. A widow who is not cleansed cannot come near other people because it is believed that she has impurity and the spirit of death.

‘*The things the community does that force a widow to be cleansed are they cannot come near you. They cannot interact with you because they are fearful. You have a bad omen[impurity] because your husband died. They avoid you and keep their distance.' (Participant 7)*

*The children are not allowed to see you. You cannot eat with them. You cannot greet your grandchildren. I went through a very difficult experience (Participant 19)*

Eight widows detailed being restricted from entering other people's homes or fetching water because sexual cleansing had not yet occurred.

‘*They force you that you must be cleansed so that you can interact with people. They do not want you to go near people – interact with them. Like in my case when my husband died and a father-in-law died, they did not want me to meet with his wife, and yet we live in the same compound. We could not go and fetch water from the same river. We cannot fetch water from the same stream before I am cleansed, and she is cleansed. So, restrictions were forced on us.' (Participant 26)*

#### To be eligible to remarry

Widows reported living with the man who cleansed them, and this was referred to as widow inheritance. Although widow inheritance and sexual cleansing were terms not clearly defined and used interchangeably, many of the widows did not live with their cleansers after the ritual was complete. A widow is cleansed to become a wife in the home as described in the following excerpt:

*A widow is cleansed because it is a tradition that was set a long time ago. They [community members] believe that if you are cleansed then you are a wife in the home. After your husband dies you are cleansed to make you a wife in the home. If you are not cleansed they know you will leave and go and live elsewhere. If you are cleansed you become their wife. (Participant 24)*

The eligibility to re-marry was contingent on the age of the children and the participant's age when widowed. Widows who had young children upon the death of their husband, and were considered by in-laws and community members as young and therefore having the ability to have more children, described an obligation per tradition to go through sexual cleansing. This is in contrast to the older widows with adult children that were perceived to be beyond the child bearing age. Sexual cleansing and widow inheritance prohibited the widow from having sexual relationships. Young widows were sexually desirable and sexual cleansing was prescribed to them by in-laws and community members because the widow could have additional children as illustrated in the excerpt below:

*A widow is cleansed because of the myths the Luos have – the family can say their brother has left a young wife who may still want to get children. The Luos do not think that you can be satisfied with 2 or 3 children like long time ago, it would be said that our brother has left a wife so she is given somebody or forced to be with somebody. There are families who will force you to live with somebody. (Participant 25)*

Also, financial support for the widow and her children was a justification for sexual cleansing and widow inheritance as depicted in the quote below:

*During that time, I had to be cleansed because I was young...I had four children. People felt regretful that I was left with a very young family and wondered how I would manage. (Participant 10)*

### Enforcement of sexual cleansing

The individuals within the community, especially the widow's family, in-laws, and children enforced sexual cleansing. Sexual cleansing was enforced by inciting the children, treating the widow with hostility, and restricting an uncleansed widow from interacting with other community members and building a homestead. The enforcement was in-place to uphold tradition and prevent the death of the children.

#### Role of the widow's family

Four participants discussed the role of their immediate family in sexual cleansing. Examples included sending the widow who has refused to be sexually cleansed back to her matrimonial home because the father-in-law talked to the widow's family that sexual cleansing must occur to honor tradition. Other examples include the sister hosting the widow for a period of time if she has received a lot of pressure from in-laws to be sexually cleansed but does not want to go through the ritual.

“*Widow cleansing is a community's tradition. The community and brother in-law forces widows to be inherited especially if you have children, even if you've refused.” (Participant 2)*

#### Role of in-laws

The role of in-laws in the sexual cleansing ritual was observed in the selection of cleansers either by performing sexual cleansing themselves, introducing the cleanser, or paying for a hired cleanser. They also incited the widow's children and treated her with hostility if she resisted being sexually cleansed. If the brother-in-law is not performing the sexual cleansing ritual, the cleanser is brought by the brother-in-law as a guest to the widow's household. Three of the participants mentioned that they were unaware that the guest introduced by the brother-in-law was the one to sexually cleanse her because they came unannounced. Two other participants mentioned that a cleanser was forcefully brought to the widow by her husband's relatives so that the cleanser could take the place of her late husband.

The widow is coerced by her in-laws into sexual cleansing when they incite the children against her and tell her she cannot visit the households of other relatives and/or build a new house in the father-in-law's compound. Only one participant described that the widow has the power to choose not to be sexually cleansed if she has supportive relatives who honor her decision.

‘*It depends on the home where she's married, if it's a home where inheritance is a must, she will be chased away but if it's a home where she's left free, she will just do what she wants.' (Participant 9)*

More than half of the participants used the term *force* to describe the demands communicated by in-laws to enforce sexual cleansing. All of the widows had children and according to tradition, they were cleansed because of the children. Both male and female children are incited to believe that their uncleansed mother will kill them. The in-laws insisted that she had to be cleansed because she is impure, and the widow was not permitted to mingle freely, eat, and live with the children in the home. The male children, especially, were most persuaded because if their mother was uncleansed because they were unable to build a home and consummate their marriages as described in the following excerpt:

*It was difficult for me but I have sons, so my brother-in-law said my sons cannot build the homestead if I am not cleansed. I was forced into it and I accepted it because of my sons could be able to build and consummate their marriages if I was cleansed. (Participant 2)*

The collusion could lead to children abandoning their mother who is not cleansed. Children abandoning their mother was mentioned as withholding financial support if she is not cleansed.

‘*They tell them that if your mother is not cleansed, you will die. Your mother wants to kill you or your mother is eating with you. They incite them, even the daughters are incited that your mother will bring them death.' (Participant 25)*

Several widows alluded to the fear of HIV in the community as it relates to sexual cleansing when brothers-in-law refused to cleanse because they are uncertain of the cause of death of the widow's husband. Instead, the brother-in-law brought another brother-in-law from the clan or an outsider known to the family or he will instruct the widow to look for a cleanser. Some widows described the challenges of searching for a cleanser as instructed by in-laws because if unable, she cannot return and/or stay in the home.

#### Role of children

The role of the widow's children was one of the main findings as to why sexual cleansing occurred. Participants explained that the uncleansed widow is blamed for the death of the children in the family and community. Children had a role in influencing their mothers to go through sexual cleansing. The children were the widow's children, but also her grandchildren and other children in the community. A few widows mentioned that if they were not cleansed of impurity or evil spirits, the children would be unhappy, hold grudges, and express worry that they would die as demonstrated in the following quote:

*She was not cleansed and she is touching your children with a bad omen [evil spirit]. She is the one who has caused your child's death. So, the children have a grudge against you that you have caused their child's death. (Participant 8)*

*They tell them that if your mother is not cleansed, you will die. Your mother wants to kill you or your mother is eating with you. They incite them, even the daughters are incited that your mother will bring them death. (Participant 21)*

## Discussion

The current study adds to the limited existing literature examining the social and cultural components, and significance of sexual cleansing (8, 9). Study findings provide insight into how the practice is socially constructed and reinforced in practicing communities. The practice is a social construct because its meaning is created through social interactions based on the understanding and beliefs about death. According to study findings, family especially in-laws and community members enforce sexual cleansing because of the belief that the widow is impure with evil spirits. Current studies have illustrated that the sexual cleansing ritual removes impurity and evil spirits tied to the dead, and its purpose is to protect the family and community members (4, 8, 9, 11, 12). Impurity is removed through sexual intercourse, a mandatory component of the sexual cleansing process, which also protects them from further harm such as illness (19). Individuals who do not act accordingly based on the belief system are shunned and ostracized (8).

The perceptions toward sexual cleansing are maintained and reinforced through traditions. One of the key findings is that widows were sexually cleansed because of the demands that are placed on her by in-laws, immediate family members, and community members. These demands are in place to fulfill the Luo traditions and to protect the children, extended families, and the community from evil spirits. Norms and traditional customs of sexual cleansing are prescribed and are non-negotiable because the widow is married into patrilocal and patriarchal family structures (13). Study findings show that the social pressure to honor tradition based on the cultural expectations that a widow must be sexually cleansed reinforces the practice by restricting the widow's ability to interact with her children, enter other people's homes, complete daily activities such as fetching water, and build/or repair a grass-thatched house. These social pressures and restrictions are forms of social control to create a normative compliance to abide by the norms within the community. The Luo are organized by patrilineal and patrilocal societies (27), where the woman is the wife of the clan because she relocates and marries into the family. Common features of systems of masculine hegemony, or patriarch, are the social construction of men as superior to women, as women's providers and protectors, responsible for ensuring their welfare and guarding their sexuality, and of intimate partner violence as an aspect of masculinity and a legitimate means for men to control and instruct women (28). Male children are highly prized and a family without a male child holds low status in community (20). For men in patrilineal societies, the primary practices associated with the attainment of manhood and adulthood were provision of land to a wife or wives for subsistence farming, establishing a household, and siring children, in addition to gaining authority over one or more women through bride wealth-based marriage (29). This is exemplified in the practice of sexual cleansing and inheritance, as it is only widows that are subjected to sexual cleansing. Due to this, the bereaved woman is required to fulfill the rituals of sexual cleansing (13). This demonstrates further that the practice of sexual cleansing is a social construct because it is the product of the interactions between the widow and others in the community (30).

Results of this study show that sexually cleansed widows were forced into the cultural practice. Based on the perception and compliance to the ritual as tradition, widows were excluded in the decision making of whether or not to be cleansed because brother in-laws brought cleansers unannounced and introduced them as guests to the widow. Some participants reported that they were locked in the house with the cleanser by the brother in-law to ensure that the cleansing occurred. Further, findings from this study suggest that sexually cleansed widows were forced into the cultural practice. These findings suggest that sexually cleansed widows were opposed to the practice which is consistent with the findings of Cruz et al. (4). The study by Cruz et al. (4) found that the majority of their participants (189 women and 170 men) had unfavorable perspectives to sexual cleansing and viewed it as outdated practice in present society which undermined women's rights and did not represent equality of treatment between men and women. These findings illustrate unequal gender power relations and the subservient position of women to men. The practice can be attributed to the oppression and subordination of women to men because cultural demands to remove impurity in most African societies are often placed on the widow and not the bereaved man (1, 4). The social constructionism theory poses that the taken-for-granted assumptions, referred to as the aspects of social life that are unquestioned and widely accepted are in fact connected to the interest of those in power, and are therefore reinforced in the interest of the dominant social group (30, 31). Thus, the cultural practice of sexual cleansing is a justification of oppression that is labeled as tradition.

Sexual cleansing is institutionalized and reinforced in the Luo community through restrictions placed on widows. Among the Luos, it is customary that the woman relocates to where her husband's family is located because it is a patrilineal culture (Philip et al., 20159). A widow is forced into wife inheritance and consequently sexual cleansing to remain on her late husband's property. Thus, to remain on the property, the widow must move from a stable structure to a grass-thatched house, a temporary structure where the sexual cleansing will be observed. These structures are poorly built, unstable, and bound to destruction over time due to pests and weather conditions, the widow must reside her until she is cleansed. Furthermore, social restrictions were placed on the widow that she cannot build a house without the assistance of a brother-in-law. These restrictions were also applicable to her children especially sons as they cannot construct a house without their mother being sexually cleansed.

The means by which sexual cleansing is socially constructed can be observed in the manner in which widow is treated compared to a married woman. Widows reported being treated negatively because her husband is deceased which is very different from how a married woman would be treated. This mal-treatment in form of abuse and ostracization is what eventually leads the widow to accept sexual cleansing against her wishes (8, 10). Also, widows are sexually cleansed because her status is tied to her proximity to males to improve her status, value, and treatment in the community. In some patrilineal societies, the status, identity, and worth of women is tied to her husband and his family (18). This conclusion is supported by the findings of this study where the widows reported that they were regarded and treated as “a nobody” because they had no husband. Overall, the findings of this study are consistent with current literature on wife inheritance and sexual cleansing (4, 8–12).

The findings from this study demonstrate that the continued perpetuation of sexual cleansing reinforces the patriarchal system which subjugates women in society and this has several implications for the health and wellbeing of widows in the Luo community and women globally. Widowhood represents not only the spousal loss and personal grief but the loss of social status, financial strain and social isolation. For many women in low-income countries in Africa and Asia, cultural beliefs surrounding death and the ill-treatment of widows is associated with dire negative emotional and psychological effects (6; (7)). Sexual cleansing presents additional physical health consequences such as an increased risk of HIV/AIDS and other sexually transmitted diseases, as well as trauma from physical abuse. Also, the fact that women are forced to fulfill these rituals highlight the subversion of these women's human rights and autonomy. Women who may be opposed to this practice are compelled *via* societal pressure to undergo cleansing. Further, sexual cleansing threatens widows' economic independence as social restrictions prevent them from re-building their lives after the loss of their spouse without the consent and support of a brother-in-law. Also, there is the concern of the socialization of young girls on women subordination with the practice of sexual cleansing. In communities that uphold this practice, family members and the society at large act as agents of socialization as an uncleansed widow is considered a disgrace to herself and to her family (8). The social construction of sexual cleansing relies on this socialization process ensuring that these norms and traditions are reproduced across generations.

The negative physical, mental, and social health implications of widow sexual cleansing highlight the need for a multi-level integrated interventions to eliminate the practice of sexual cleansing. These interventions should focus on increasing and strengthening widows' knowledge of the harms of the practice and human rights at the individual level as well as a community level educational intervention. Cultural norms and practices are not static and can be transformed and re-invented, evolving with changing times (32). Research shows the increasing adoption of alternative rituals to widow sexual cleansing with the advent of the HIV/AIDS epidemic (33). These symbolic cleansing are performed without any actual sexual intercourse and considered viable options to completing cultural customs (34). The modification of the ritual in the Luo community is imperative. However, as Banda and Kunkeyani (35) have pointed out, it is important that changes to cultural practices be socially transformative, as they touch on the restructuring of belief systems and the re-negotiation of socio-cultural contexts associated with sex and power relations. Efforts geared toward increasing awareness of alternative rituals should be accompanied with socio-economic empowerment initiatives aimed at fostering self-reliance among widows (36).

This study is not without limitations. As this study focused on the perspectives of widows that have undergone sexual cleansing, the analysis was limited to their experiences. The perspectives of widows who may have been able to resist the practice were not included in this study. Future research may build on the findings of this study by investigating strategies employed to evade the ritual. Available research suggests that a woman's educational and economic status, to some extent, grants the privilege to accept or reject which widowhood rites to perform (37). Future research can also assess the role of poverty in the continued practice of sexual cleansing of women in the Luo community. It is possible that educated non-poor women are not subjected to fulfilling the ritual and are therefore able to resist the pressure for cleansing. Additionally, this analysis lacks the perspective of the male in-laws as well as the hired cleansers. Engaging men is imperative to understand the continued perpetuation of the practice and can be instrumental to eliminating it (38). Male perspectives of this cultural practice can be assessed in future research studies.

## Conclusion

This analysis is one step into an important area of research that examines the viewpoints of widows. The results of this study point to a further need to detail the social, emotional, financial, and health implications aside from HIV that are common to widows who are sexually cleansed. Such studies would bring attention to the impact of sexual cleansing on widows that will inform the community to ultimately decide whether or not the practice ought to continue, as well as identify prominent individuals and community-based organizations in mobilizing the eradication of the practice.

## Data availability statement

The original contributions presented in the study are included in the article/supplementary material, further inquiries can be directed to the corresponding author/s.

## Ethics statement

The studies involving human participants were reviewed and approved by Amref Health Africa Ethics and Scientific Review Committee (ESRC) and St. Catherine University IRB. The patients/participants provided their written informed consent to participate in this study.

## Author contributions

LM conceptualized the study. LM, EM, and MH contributed to the data analysis and interpretation. LM, EM, MH, NO, and BY contributed to manuscript preparation and revision. All the authors critically revised the manuscript and approved the submitted version.

## Funding

This project was funded by an Academic Professional Development Committee (APDC) Faculty Research & Scholarly Activities Grant from St. Catherine University.

## Conflict of interest

The authors declare that the research was conducted in the absence of any commercial or financial relationships that could be construed as a potential conflict of interest.

## Publisher's note

All claims expressed in this article are solely those of the authors and do not necessarily represent those of their affiliated organizations, or those of the publisher, the editors and the reviewers. Any product that may be evaluated in this article, or claim that may be made by its manufacturer, is not guaranteed or endorsed by the publisher.

## References

[1] Sossou MA. Widowhood practices in West Africa: The silent victims. Int J Soc Welf. (2002) 11:201–9. 10.1111/1468-2397.00217

[2] Fiasorgbor D. Widowhood Rite: an infringement on the rights of widows in Bongo. Int. J. Dev. Soc. (2018) 7:1–8. 10.11634/216817831504951

[3] Kotzé E Els L Rajuili-Masilo N. “Women… mourn and men carry on”: African women storying mourning practices: A South African example. Death Stud. (2012) 36:742–66. 10.1080/07481187.2011.604463 24563939

[4] Cruz GV Mateus A Dlamini PS. HIV prevention: mapping mozambican people's views on the acceptability of the widow's sexual cleansing ritual called Pita-kufa. BMC Int Health Hum Rights. (2018) 18:e0177. 10.1186/s12914-018-0177-z 30236108 PMC6149074

[5] Ajayi LA Olanrewaju FO Olanrewaju A Nwannebuife O. Gendered violence and human rights: An evaluation of widowhood rites in Nigeria. Cogent Arts Human. (2019) 6:1676569. 10.1080/23311983.2019.1676569

[6] Djankpa GB. Effects of Widowhood Rites on the Psychological Distress and Life Satisfaction of Konkomba Widows in the Saboba District, Northern Ghana (Doctoral dissertation, University of Cape Coast) (2021).

[7] Miriam Reinette BO. Widowhood practices in south-eastern Nigeria: An aspect of women exclusion in leadership, governance, and development. GOUNI J Manage Soc Sci. (2016) 3:35–52. http://journal.gouni.edu.ng/index.php/fmss/article/view/35

[8] Ayikukwei R Ngare D Sidle J Ayuku D Baliddawa J Greene E. Social and Cultural Significance of the Sexual Cleansing Ritual and its Impact on HIV Prevention Strategies in Western Kenya. Sex Cult. (2007) 11:32–50. 10.1007/s12119-007-9010-x

[9] Ayikukwei R Ngare D Sidle J Ayuku D Baliddawa J Greene J. HIV/AIDS and cultural practices in western Kenya: The impact of sexual cleansing rituals on sexual behaviours. Cult Health Sex. (2008) 10:587–99. 10.1080/13691050802012601 18649197

[10] Perry B Oluoch L Agot K Taylor J Onyango J Ouma L. Widow cleansing and inheritance among the Luo in Kenya: The need for additional women-centered HIV prevention options. J Int AIDS Soc. (2014) 17:e19010. 10.7448/IAS.17.1.19010 24973041 PMC4074366

[11] Agot K Vander Stoep A Tracy M Obare B Bukusi E Ndinya-Achola J. Widow inheritance and HIV prevalence in Bondo District, Kenya: Baseline results from a prospective cohort study. PLoS ONE. (2010) 5:e14028. 10.1371/journal.pone.0014028 21103347 PMC2984493

[12] Ambasa-Shisanya C. Widowhood in the era of HIV/AIDS: A case study of Siaya District, Kenya. SAHARA: J Soc Aspects HIV/AIDS Res Alliance. (2007) 4:606615. 10.1080/17290376.2007.9724882 18071612

[13] Okech A. Widow Inheritance and Contested Citizenship in Kenya. London: Routledge (2019). 10.4324/9780429022715

[14] Mabumba ED Mugyenyi P Batwala V Mulogo EM Mirembe J Khan F. A.. Widow inheritance and HIV/AIDS in rural Uganda. Trop Doct. (2007) 37:229–31. 10.1258/004947507782332955 17988488

[15] Kalinda T Tembo R. Sexual Practices Levirate Marriages in Mansa District of Zambia. (2010). Available online at: https://www.researchgate.net/publication/292636348_Sexual_practices_and_levirate_marriages_in_Mansa_District_of_Zambia (accessed March 23, 2021).

[16] Warria A. Girls' innocence and futures stolen: the cultural practice of sexual cleansing in Malawi. Child Youth Serv Rev. (2018) 91:298–303. 10.1016/j.childyouth.2018.06.011

[17] Malungo J. Sexual cleansing (Kusalazya) and levirate marriage (Kunjilila mung'anda) in the era of AIDS: changes in perceptions and practices in Zambia. Soc Sci Med. (2001) 53:371–82. 10.1016/S0277-9536(00)00342-7 11439820

[18] Luginaah I Elkins D Maticka-Tyndale E Landry T Mathui M. Challenges of a pandemic: HIV/AIDS-related problems affecting Kenyan Widows. Soc Sci Med. (2005) 60:1219–28. 10.1016/j.socscimed.2004.07.010 15626519

[19] Oluoch EA Nyongesa WJ. Perception of the rural Luo community on widow inheritance and HIV/AIDS in Kenya: towards developing risk communication messages. Int J Bus Soc Sci. (2013) 4:108.

[20] Olang'o CO Nyambedha E Aagaard J. Practice of sumo kodhi among the Luo and implications for HIV transmission in western Kenya. Afr J AIDS Res. (2014) 13:383–91. 10.2989/16085906.2014.985238 25555104

[21] Bicchieri C. The Grammar of Society: The Nature and Dynamics of Social Norms. Cambridge: Cambridge University Press (2006). 10.1017/CBO9780511616037

[22] Crossley N. Key Concepts in Critical Social Theory/Nick Crossley. London: SAGE (2005). 10.4135/9781446220702

[23] Varol N Fraser IS Ng CH Jaldesa G Hall J. Female genital mutilation/cutting–towards abandonment of a harmful cultural practice. Austr New Zealand J Obstetr Gynecol. (2014) 54:400–5. 10.1111/ajo.12206 24801568

[24] Vissandjée B Denetto S Migliardi P Proctor J. Female Genital Cutting (FGC) and the ethics of care: community engagement and cultural sensitivity at the interface of migration experiences. BMC Int Health Hum Rights. (2014) 14:13. 10.1186/1472-698X-14-13 24758156 PMC4012131

[25] Siaya County Government. Siaya County Spatial Plan 2018-2028. Siaya, Kenya: Siaya County Government (2018).

[26] Hsieh H Shannon SE. Three approaches to qualitative content analysis. Qual Health Res. (2005) 15:1277–88. 10.1177/1049732305276687 16204405

[27] Nyambedha EO Aagaard-Hansen J. Practices of relatedness and the re-invention of duol as a network of care for orphans and widows in western Kenya. Africa. (2007) 77:517–34. 10.3366/afr.2007.77.4.517

[28] Schuler SR Lenzi R Badal SH Nazneen S. Men's perspectives on women's empowerment and intimate partner violence in rural Bangladesh. Cult Health Sexual. (2018) 20:113–27. 10.1080/13691058.2017.1332391 28594292 PMC5718933

[29] Mojola SA. Providing women, kept men: doing masculinity in the wake of the African HIV/AIDS pandemic. Signs. (2014) 39:341–63. 10.1086/673086 25489121 PMC4257489

[30] Weinberg D. Contemporary Social Constructionism. Philadelphia: Temple University Press (2014).

[31] Galbin H. An introduction to social construction. Soc Res Rep. (2014) 26:82–92. 10.5860/choice.33-3018

[32] Esho T Van Wolputte S Enzlin P. The socio-cultural-symbolic nexus in the perpetuation of female genital cutting: a critical review of existing discourses. Afrika Focus. (2011) 24:53–69. 10.1163/2031356X-02402005

[33] Saguti E,. Alternative Rituals of Widow Cleansing in Relation to Women's Sexual Rights in Zambia. (Master Thesis). (2017). Available online at: https://researchspace.ukzn.ac.za/handle/10413/14323

[34] Nyanzi S Nassimbwa J Kayizzi V Kabanda S. ‘African sex is dangerous!' Renegotiating ‘ritual sex' in contemporary Masaka District. Africa. (2008) 78:518–39. 10.3366/E0001972008000429

[35] Banda F Kunkeyani TE. Renegotiating cultural practices as a result of HIV in the eastern region of Malawi. Cult Health Sex. (2015) 17:34–47. 10.1080/13691058.2014.944569 25138154

[36] Momanyi JN Getui NM Ojore AO. Socio-economic empowerment of widows for sustainable self-reliance in Kajiado West Sub-County, Kenya. J Afr Interdisc Stud. (2021) 5:24–46. https://kenyasocialscienceforum.files.wordpress.com/2021/05/pdf-momanyi-et-al-socio-economic-empowerment-of-widows-in-kajiado-kenya.pdf

[37] Genyi GA George-Genyi ME. Widowhood and Nigerian womanhood: Another context of gendered poverty in Nigeria. Res Human Soc Sci. (2013) 3:68–73. https://core.ac.uk/download/pdf/234673507.pdf

[38] Flood M. Harmful traditional and cultural practices related to violence against women and successful strategies to eliminate such practices–working with men. in United Nations Economic and Social Commission for Asia and the Pacific Expert Group Meeting–Strategies for Implementing the Recommendations From the Secretary-General's Study on Violence Against Women with Particular Emphasis on the Role of National Machineries, Bangkok, Thailand (2007).

